# Origin of the Autophagosome Membrane in Mammals

**DOI:** 10.1155/2018/1012789

**Published:** 2018-09-24

**Authors:** Yun Wei, Meixia Liu, Xianxiao Li, Jiangang Liu, Hao Li

**Affiliations:** ^1^Xiyuan Hospital of China Academy of Chinese Medical Sciences, Beijing 100091, China; ^2^Department of Oncology, Air Force General Hospital, Beijing 100142, China

## Abstract

Autophagy begins with the nucleation of phagophores, which then expand to give rise to the double-membrane autophagosomes. Autophagosomes ultimately fuse with lysosomes, where the cytosolic cargoes are degraded. Accumulation of autophagosomes is a hallmark of autophagy and neurodegenerative disorders including Alzheimer's and Huntington's disease. In recent years, the sources of autophagosome membrane have attracted a great deal of interests, even so, the membrane donors for autophagosomes are still under debate. In this review, we describe the probable sources of autophagosome membrane.

## 1. Introduction

Macroautophagy (henceforth known as autophagy) is a nonselective “self-eating” process that maintains cellular homeostasis, manages stress responses, and controls large proteins and cytoplasmic components quality by eliminating defective or superfluous molecules/structures such as misfolded proteins, damaged mitochondria, excessive peroxisomes, ribosomes, and invading pathogens. Autophagy, which can be induced by exogenous stimulations, such as nutrient starvation, endoplasmic reticulum (ER) stress, rapamycin, vitamin D3, and IFN-*γ* treatment, provides a source of nutrition and emergy during periods of stress to promote healthy cell homeostasis and synaptic function. Dysfunction of autophagy is a misregulated process in various neurodegenerative diseases, different types of cancer, autoimmune diseases, and uncontrolled infections characterized by the accumulation of protein aggregates, degradation of intracellular pathogens, and clearance of aging organelles, including Alzheimer's disease, Huntington's disease, Parkinson's disease, and amyotrophic lateral sclerosis.

The function of autophagy relies on the formation of double- or multimembrane vesicles named autophagosome (AP), which plays a key role in cell homeostasis through engulf cargo including damaged mitochondria (mitophagy) and protein aggregates. The activity of autophagy is modulated primarily by the size and number of APs. The production/accumulation of APs subsequently unfuse to lysosomes (or accumulation of APs) directly induces cellular toxicity under the condition of various stress conditions, such as oxidation and toxic protein aggregation, and this process may be implicated in the pathogenesis of neurodegenerative diseases, tumorigenesis, and infections, among others. The acidic pH and enzymatic action of hydrolases within the lysosome lead to the breakdown of the internal membranes of APs as well as the APs' contents. So far, however, the origins of the autophagosomal membrane and the molecular mechanisms of AP formation are still unknown.

Here, we organize and summarize the papers in this issue according to four focus areas: morphology, formation, function, and source of AP.

## 2. Morphology of Autophagosome

As the double- or multimembrane organelle, the AP in the cytoplasm is a hallmark and the key initial event of autophagy. Sometimes double-membrane structure which contains part of cytoplasmic components also can be judged as AP. The size and number of APs may be separately regulated by different subgroups of autophagy-related genes (ATGs) proteins and the members of the ATG8/ light chain 3 (LC3) protein family. This makes sense considering that modulating AP size may affect primarily cargo selectivity, while regulating the number of APs could be carried out to regulate mostly autophagy flux. In mammals, the time of the AP formation takes 5-10 min [1]. The time courses of starvation decide the extent of AP expansion. The amount of ATG9 protein correlates with the numbers of autophagic bodies; that is, ATG9 levels determine the number of APs. Nitrogen-starvation induces APs range from 300 to 900 nm in diameter, larger than most other vesicles in the cell. During nonselective autophagy, premature closure of the phagophore results in smaller AP.

Another study has found that the size of AP may also depend on specific cargo [2], which can range from proteins to intracellular bacteria (0.06 to 0.2 *μ*m) [3]. The size and formation of APs are regulated by the steady-state level of microtubule-associated protein LC3 [4], but the regulatory mechanism of this protein is not understood. The size of APs is likely determined by distinct autophagic steps. Frist, APs can expand by the addition of membrane to form isolation membranes during the early stage of autophagy. Second, APs may grow by fusion with endosomes and lysosomes at the late stage of autophagy, though endosomal/lysosomal fusion is not sufficient for proper autophagosomal growth. Besides, the extent of AP expansion is mainly dependent on the time course of induction condition, such as starvation. The reduction of the cellular level of ATG8, which anchored to the surface of APs, results in smaller AP compared with a wild-type strain, but the number of AP is the same as that of the wild type. Meanwhile, the AP is a highly dynamic organelle and its proteome differs if cells are under the conditions of starvation or basal macroautophagy when blocked with concanamycin A.

## 3. Formation of Autophagosome

**Figure 1:**
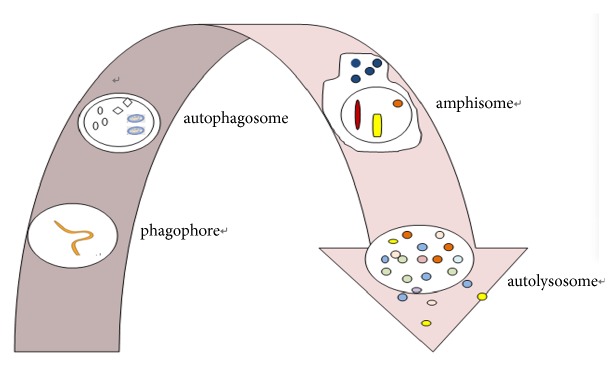
Overview of the autophagy process.

AP formation is a complex series of discrete events which is mediated and controlled by a large number of proteins, but the process is poorly understood. Essentially, APs are formed by the induction, expansion (the phagophore/isolation membrane and the omegasome), vesicle completion (the AP), fusion (the amphisome), and degradation (the autolysosome/lysosome) [5], as shown in Figure 1. In the brain, AP biogenesis occurs distally in a constitutive process at the neurite tip which is far away from the nucleus in a microtubule- and dynein-dynactin motor complex-dependent manner. Various stress conditions, such as starvation, oxidation, and toxic protein aggregation, can accelerate the biogenesis of APs and degradation of lysosome. A mature AP is generated when the isolation membrane closes, and then the mature APs fuse with the vacuole/lysosome, where the contents are degraded and the products recycled to the cytosol for reuse. Nearly 40 ATGs have been identified and most of them are conserved across higher eukaryotes, but only parts of ATGs are directly associated with mammalian AP biogenesis, as shown in Table 1. The homotypic fusion of ATG16-positive and LC3-negative AP precursors is a critical regulatory step in AP biogenesis. As the conjugated form of LC3, LC3-II is associated with the outer of the autophagosomal membranes following completion and remains with the AP until fusion with the lysosomes [6]. As an aside, elevated levels of LC3-II generally correlate with the accumulation of APs in the cell but so not indicate an increase in AP biogenesis.

There are reports that the ULK1/ATG1-ATG13-FIP200-ATG101 protein kinase complex, the VPS (vacuolar protein sorting) 34 (VPS34) complex, the ATG9 trafficking system, ATG5-ATG12-ATG16L1 complex, the two ubiquitin-like proteins ATG12 and ATG8/LC3, and their conjugation systems all have been found which lead to formation and expansion of the phagophore, which eventually seals to form the complete AP. Meanwhile, AP formation is highly inducible. Amino acid can induce autophagy and the protein kinase complex TORC1/mTORC1 suppresses AP formation in nutrient-rich conditions. Actin protein also is necessary for starvation-mediated autophagy through the Arp2/3 complex and WHAMM and actin depolymerization participates in the formation of APs at a very early stage rather than in the maturation steps. In addition, the deconjugation of ATG8–phosphatidylethanolamine (PE) is also required for efficient AP biogenesis for optimal phagophore expansion.

## 4. Function of Autophagosome

Autophagy is an evolutionarily conserved cellular process to maintain energy homeostasis. As the marker of autophagy, the dynamics and functions of APs remain robust in the mouse model of neurodegenerative disease, but AP flux is not increased even as protein accumulates along the axon [23]. Currently, stimulation of APs synthesis is often used to enhance autophagy to alleviate aggregation toxicity of protein in neurodegeneration and aging. Retrograde transport of APs might play a role in neuronal signaling processes, promoting neuronal morphological complexity and preventing neurodegeneration. Autophagy can promote infection by picornaviruses, such as poliovirus and coxsackieviruses, just because APs provide sites for replication. As the double-membrane vesicles, the compositions of the outer and inner AP membranes seem to be quite different [24]. So, there are different roles of different membranes: the inner autophagosomal membrane in charge of cargo sequestration and the outer autophagosomal membrane in charge of fusion with the lysosomal membrane.

Meanwhile two characteristics make APs a unique type of cellular transport carrier. First, two lipid bilayers surround the cargo and second, these giant vesicles generally have an average diameter of approximately 700 nm, which can further expand to accommodate large structures such as cellular organelles and bacteria [25]. But accumulation of APs causes cytotoxicity; especially in certain stress conditions the excessive APs subsequently unfused to lysosomes directly induce cellular toxicity independent of apoptosis and necroptosis, and this process may be implicated that AP is a hallmark of neurodegenerative disorders including Alzheimer's and Huntington's disease or amyotrophic lateral sclerosis [26, 27].

## 5. Source of Autophagosome Membrane

Because autophagy is an unselective bulk degradation pathway, the specific membrane origin of all APs remains obscure, though morphological features of APs are basically common to conventional and alternative autophagy. Different from yeast and plant cells, there is no preautophagosomal structure (PAS) in mammalian cells. The endoplasmic reticulum exit sites (ERES), mitochondria, ER-mitochondria contact sites, ER-Golgi intermediate compartment (ERGIC), Golgi apparatus, and plasma membrane (PM) have been suggested to supply lipids to the growing isolation membrane in mammalian cells, but the exact mechanism mediating this process remains ambiguity. It is possible that there are different membrane sources dependent on the cell type, growth conditions, physiological conditions, and so on, as shown in Table 2.

## 6. ER-Mitochondria Contact Sites [28]

ER-mitochondria contact sites have gained a lot of attention recently. In 1952, Wilhelm Bernhard first reported the ER-mitochondria contact sites in rat liver by electron micrographs [29]. The ER-mitochondria contact sites are essential for several key cellular processes, such as calcium homeostasis, lipid metabolism, autophagy regulation, mitochondrial dynamics [30], cell survival to energy metabolism, and protein folding [31]. The correct maintenance of the ER-mitochondria interface is a critical part of the autophagic process; meanwhile the emergence of phagophores and the sites of contact between the ER and mitochondria have surprising correlation. Upon starvation, components of the autophagy-specific class III PI3K (ATG14L, Vps34, Beclin1, and Vps15) all accumulated in the mitochondria-associated membranes (MAMs) fraction and probably recruited to ER-mitochondria contact sites upon autophagy induction, and the autophagosome-formation marker ATG5 also localizes at the ER-mitochondria contact site until AP formation is complete. And AP formation is significantly suppressed in mitofusin 2-knockout cells, in which the ER-mitochondria contact sites are disrupted [32].

## 7. Endoplasmic Reticulum Exit Sites

As the largest organelle in the cell, the ER is in close proximity with other endomembrane compartments because of its membrane lipids and proteins synthesis and outward transports, establishing membrane-membrane contact sites (MCS) that facilitate signaling events, modulate dynamic organelle processes, and exchange the lipids and ions. ERES, the specialized regions of the ER where COPII transport vesicles are generated, are thought to be spatially, physically, and functionally linked to APs. Early autophagic structures tightly associate with the ER membrane due to the presence of omegasome subdomain positive for double FYVE-containing protein 1 (DFCP1), a phosphatidyl-inositol-3-phosphate (PI3P) binding protein; meanwhile the omegasome isolation membrane and AP phagophore probably are all derived from the ER [34]. Sandra Maday [45] also thought that APs are generated at DFCP1-positive subdomains of the ER in the distal end of the axon, distinct from ER exit sites in primary neurons. Studies have found that [46] lysosome membrane-associated protein-2 (LAMP-2), as a heavily glycosylated type-1 membrane protein, must be critical for translocating syntaxin-17 (STX17) to autophagosomal membranes. Sanchez-Wandelmer et al. [47] have found that ERES are core elements in the formation of isolation membranes and ER associates with the extension of isolation membranes or phagophore in mammalian cells by electron tomography. However, contradictory data emerged indicating that only 30% of all APs are associated with the ER or specialized regions of the ER [36].

## 8. Mitochondria

The mitochondria are the most important organelle in determining fundamental metabolic activities, iron and calcium homeostasis, and signal transduction of various cellular pathways. Mitochondrial dysfunction and dysregulation may lead to many human maladies, including cardiovascular diseases, neurodegenerative disease, and cancer. A number of reports suggest that [32] there is a connection between mitochondrial outer membrane lipids, proteins, and AP formation. It is suggested that the outer mitochondrial membrane donates a flow of lipids and membrane proteins to the AP. The early autophagy protein ATG5 and the autophagosomal marker LC3 translocate to puncta localized on the outer membrane of mitochondria following starvation, suggesting that mitochondria plays a central role in starvation-induced autophagy. Meanwhile, researches have found mitochondrial membrane donation to AP formation in both basal (in the presence of serum and vehicle) and drug-induced autophagy in human breast cancer cell line, other than being engulfed by the forming AP [48]. In other studies the authors also show that the connection between ER and mitochondria is crucial because in its absence, starvation-induced APs are not formed [32]. The counterargument is that mitochondria have nothing to the source of AP membranes, because mammalian ATG9 is found only to localize to the trans-Golgi network (TGN) and late endosomes, but not to mitochondria [49], while ATG9-containing compartments are a source of membranes for the formation and/or expansion of APs.

## 9. Golgi Apparatus

The Golgi apparatus is a major glycosylation site involved in protein and lipid synthesis, modification, and secretion. The Golgi apparatus is proposed to be a pivotal membrane source for the mammal AP formation. This result is first observed in developing invertebrate fat body cells by Locke and Collins in 1965. More researches have found that Golgi complex contributes to an early stage of autophagy [50]. At cell telophase, the Golgi structures in APs are again observed to be distributed at the cell periphery when Golgi apparatus is known to reassemble. Based on these, they proposed that Golgi apparatus is a membrane source for autophagosomal growth. As the only transmembrane ATG protein, ATG9 has been associated with the Golgi apparatus and may be involved in providing membrane for AP formation [51]. However, others argued that mammalian ATG9 (mATG9 and ATG9L1) is seen to associate with many other compartments, including recycling endosomes, early endosomes, and late endosomes [37]. It is possible that these organelles all participate in AP formation.

## 10. Plasma Membrane

The PM, which forms the barrier between the cytoplasm and the environment [52], plays critical roles in promoting virulence through mediating secretion of virulence factors, endocytosis, cell wall synthesis, and invasive hyphal morphogenesis. Meanwhile, proteins in the PM also mediate nutrient transport and sense pH, osmolarity, nutrients, and other factors in the extracellular environment. The ability of PM to contribute the AP formation may be particularly important in time of increasing autophagy in mammalian cells. The PM's large surface area might act as a massive membrane store that allows cells to experience cycles of AP synthesis at much higher rates than under basic conditions without compromising other processes. ATGs and membranes that are necessary for AP formation are originated from PM [47]. Claudia Puri et al. found that ATG16L1 associates with clathrin-coated pits, and after internalization and uncoating, the ATG16L1-associated PM becomes associated with phagophore precursors, which mature into phagophores and then APs [53].

## 11. Recycling Endosomes

In mammalian cells, the endosomal system is extremely dynamic and generates several structurally and functionally distinct compartments, namely, early/recycling endosomes (REs), late endosomes, and lysosomes. The identity of endosomes is ensured by the specific localization of regulators. REs consist of a tubular network that emerges from vacuolar sorting endosomes and diverts cargoes toward the cell surface, the Golgi, or lysosome-related organelles. REs are also implicated in AP formation. mATG9, trafficking from the PM to the REs, is essential for the initiation and progression of autophagy. A. Orsi and his research team have found that mATG9-positive structures interact dynamically with phagophores and APs. TBC1D14, a Tre-2/Bub2/Cdc16 (TBC) domain protein, regulates Tfn receptor- (TfnR-) positive REs, which are required for AP formation. As a positive regulator of AP formation, the membrane remodeling protein sorting nexin18 (SNX18) is required for regulating ATG9A trafficking from REs and formation of ATG16L1- and WIPI2-positive AP precursor membranes [54]. ATG16L1 and mATG9-positive vesicles are present in the same sites on RE, which can reduce membrane egress from the REs to increase the formation of AP. These all indicate that REs probably are membrane donator for SNX18-mediated AP biogenesis.

## 12. Endoplasmic Reticulum-Golgi Intermediate Compartment

ERGIC structures may move from ERES to the Golgi apparatus by tracking on microtubules. Membrane traffic between the ER and the Golgi is bidirectional and occurs via similar mechanisms as other MAMs. Recently, ERGIC, a membrane compartment between the ER and Golgi for cargo sorting and recycling, is proposed as another membrane source for the phagophore. At the ER-Golgi interface, coat protein complex I (COPI) vesicle buds and facilitates retrograde transport from the Golgi and ERGIC, while COPII vesicles may be the precursor of the phagophore membrane. As the donor membrane, the ERGIC is a sorting station undergoing dynamic membrane exchange with COPI and COPII vesicles [55], the later of which are supposed to a source of membrane for APs at the ERGIC. Liang Ge et al. [44] also found that generation of COPII vesicles from the ERGIC could thus be a special event for autophagy-related membrane mobilization induced by starvation-induced remodeling of ERES. Drugs that disrupt ERGIC also suppressed LC3 conjugation and LC3 dot formation.

## 13. ER-Plasma Membrane Contact Sites (ER-PMcs)

ER-PMcs are also mobilized for AP biogenesis. At present researches have identified some functions of ER-PMcs, for example, regulate Ca2+ signaling [56], and conserve lipid homeostasis. The research team of Nascimbeni AC revealed that ER-PMcs can tether to extended synaptotagmins (E-Syts) proteins, which are essential for autophagy-associated phosphatidyl-inositol-3-phosphate (PI3P) synthesis at the cortical ER membrane, and then adjust the mammal AP biogenesis [57].

According to other literatures, ATG9-containing cytoplasmic vesicles (ATG9 vesicles) [58], ER- lipid droplet (LD) contact site [59], and COPII vesicles [11]s are also considered sources of membrane to build the AP. Still others think that the ATG9-associated membranes do not fuse with APs and only regulate autophagy because of its transient structural or catalytic functions [60].

## 14. Conclusion

Lowering the accumulation of APs may be a treatment option for neurodegenerative diseases with protein aggregates, so make sure where does the AP membrane come from and how is it formed? In recent years, these questions have attracted a great deal of interests. ER, mitochondria, Golgi complex, and the plasma membrane all have been proposed as the sources of autophagosomal membranes. Of course, the origin of AP membrane is probability multisources, which has been proved by more and more researches. The diverse origins of AP membrane interact each other. These different conclusions reached by the different laboratories could be in part due to different experimental approaches and techniques used in the various laboratories. And the relative contribution of each source under any one set of conditions remains to be determined. Although distinct sources of AP membranes have been proposed, it is not clear to what extent they may be mutually exclusive or whether they may coalesce and cooperate. A lot of research is needed.

## Figures and Tables

**Table 1:** The function of ATG proteins in AP.

ATG Proteins		Features	Function in AP
Mammals	Yeast		
ULK1/2 [7]	ATG1	Serine/threonine kinase; form a complex with mATG13, FIP200 and ATG101; phosphorylated by mTORC1 and AMPK kinases	late stage of AP biogenesis

ATG2A/B [8]	ATG2	Interacts with ATG18; associates to autophagosomal membranes through lipid binding and independently from ATG9	closure of the AP membrane, late stage of AP biogenesis

ATG3[9]	ATG3	E2-like enzyme	facilitates LC3/GABARAP lipidation in highly curved membranes; curvature and maturation of AP biogenesis

ATG4A-D [1]	ATG4	cysteine protease; phosphorylation by ATG1	initial stage of phagophore formation

ATG5[10]	ATG5	conjugated by ATG12	elongation of the isolation membranes, the AP-formation marker

Beclin1	ATG6, vacuolar protein sorting (Vps)-30	conjugated by PI3KC3 and ULK	intervene at every major step in autophagic pathways, from autophagosome formation, to autophagosome/endosome maturation

ATG7	ATG7	autophagy-related E1-like enzyme	elongation of the AP membranes

LC3A/B/C, GABARAP, GATE-16, GABARAPL1/2/3 [4]	ATG8	ubiquitin-like protein; conjugates to phosphatidylethanolamine (PE)	determines the size of AP; induce membrane tethering and fusion; expansion and closure of phagophores

ATG9L1 [11]	ATG9	transmembrane autophagy-related protein	initial stage of AP formation, generate the isolation membrane

ATG10	ATG10	E2-like enzyme; catalyze or facilitate ATG5-12 conjugation	promotes autophagolysosome formation

—	ATG11	Scaffold Protein	regulates autophagosome-vacuole fusion

ATG12 [12]	ATG12	ubiquitin-like molecules; conjugates to ATG5	elongation and maturation of the phagophore membrane

KIAA0652 [13]	ATG13	Phosphorylated by (m)TORC1	later stage of autophagosome maturation

ATG14(L)/Barkor [14]	ATG14	autophagy-specific subunit	fusion of APs to endolysosomes, regulates autophagosome nucleation; the preautophagosome/autophagosome marker

ATG16L1/2 [15]	ATG16	conjugated by ATG12 and ATG5, E3‐Ubiquitin ligase‐like enzyme	elongation of AP membrane

WIPI1/2/3/4 [16]	ATG18	PtdIns(3)P-binding protein	recycle of membrane proteins from the vacuole to the late endosome

ATG19 [17]	ATG19	contains multiple ATG8 binding sites	serves as cargo receptor and directly interacts with ATG8 on the isolation membrane

—	ATG20 [18]	sorting nexin	required for efficient autophagy and membrane tubulation

—	ATG21	PtdIns(3)P-binding protein, only detected at endosomes	facilitates the recruitment of Atg8-PE to the site of autophagosome formation

_	ATG23	peripheral membrane protein	facilitates Atg9 trafficking

SNX4	ATG24 [19]	a member of the BAR domain family of proteins	inhibits the number of APs

—	ATG27 [20]	transmembrane protein	retrieval of Atg9 from the vacuole

RB1CC1/FIP200	ATG17	PI3P binding effector	
—	ATG29	Ternary complex with Atg17 and Atg31	Atg29-Atg31-Atg17 complex [21], formation a dimer with two crescents for fusion into the expanding phagophore
—	ATG31	Ternary complex with Atg17 and Atg29	

—	ATG32 [22]	outer mitochondrial membrane protein	essential for the initiation of mitophagy; facilitates mitochondrial capture in phagophores

ATG101	—	Interacts with Atg13; maintains ULK1 basal phosphorylation	interacts with the ULK1 complex via direct binding to ATG13 to induce the formation of AP

**Table 2:** The source of autophagosome membrane.

Origin	Parts of probable	Induction condition	Different stages of autophagosomes	Contribution
Mitochondria	outer mitochondrial membrane [32]	serum, or serum and amino acid deprivation [32]	phagophore expansion	the isolation membrane (also called the phagophore) expansion [1]

Endoplasmic reticulum	the rough endoplasmic reticulum [33], subdomain of the ER termed the omegasome	amino acid starvation [34], fasted animals	early stages of autophagosome formation [35]	phagophore expansion, elongation of isolation membranes [36]

Golgi	trans-Golgi network,TGN	nitrogen starvation or fasted animals	early stages of autophagosome formation [37]	phagophore formation [38] and expansion, maturation of autophagosomes [39]

Plasma membrane	ATG16L vesicle, lipids of the plasma membrane	amino acid and serum starvation, or nitrogen starvation[40]	early stages of autophagosome formation [41]	the formation of early autophagic precursors [42]

ER–mitochondria contact site	the mitochondria-associated ER membrane (MAM)	rapamycin and Torin 1 [43]	uncertain	phagophore expansion

ERGIC	ERGIC-enriched membrane	nutrient starvation [43]	generate an early autophagosomal membrane precursor [44]	trigger phagophore formation

Recycling endosomes	membrane lipids	nutrient starvation or rich medium	early stages of autophagosome formation	supply membrane lipids for autophagosome formation
